# Planning considerations of green corridors for the improvement of biodiversity resilience in suburban areas

**DOI:** 10.1186/s43065-021-00023-4

**Published:** 2021-04-06

**Authors:** Yuhong Wang, Siqi Jia, Zhe Wang, Yang Chen, Shicong Mo, N. N. Sze

**Affiliations:** 1 Department of Civil and Environmental Engineering, The Hong Kong Polytechnic University, Hung Hom, Kowloon, Hong Kong; 2 Division of Environment and Sustainability, The Hong Kong University of Science and Technology, Clear Water Bay, Kowloon, Hong Kong

**Keywords:** Green infrastructures, Pollinator insects, Highways, Habitat segmentation, Air quality

## Abstract

The world is experiencing a rapid loss in the biodiversity of pollinator insects. Habitat segmentation caused by infrastructures is one of the contributing factors. To improve the habitat connectivity of pollinator insects, it is proposed in this study to build green corridors for pollinators over linear infrastructures such as highways. In the context of suburban areas of a large city, this study examines differences in air parameters between natural environments and a roadside environment based on monitored and estimated data. Influences of different green corridor designs on floral scent dispersion are also investigated using computational fluid dynamics (CFD) modeling and simulation. It is found that, if flower plants are installed on highway overpasses, the floral scents would be better preserved as compared with those in a natural environment due to the lower concentrations of oxidative radicals in the air above highways. The stronger floral scents and their wider dispersion may help attract pollinators. Conversely, highway air contains a variety of volatine organic compounds (VOCs) that are traced to highway operations and pavements. Hence, the overall profile of VOCs in a highway environment differs from that in a natural environment. Results from CFD modeling and simulation suggest that the use of green corridors planted with flowers on the highway overpass can greatly improve the connectivity of floral scents. Hence, with proper engineering design and right combination of plant species, green corridors built on highway overpasses have the potential to facilitate pollinators to cross the road, thereby improving their habitat connectivity and resilience against declining biodiversity.

## Introduction

Resilience of infrastructures has received much attention, but the impacts of built infrastructures on the resilience of ecological environment are less examined. Recent decades have witnessed a rapid loss in biodiversity. According to the 2019 Global Assessment Report on Biodiversity and Ecosystem Service [5], 1 million out of 8 million of the total estimated number of species on Earth are under the threat of extinction. Loss in biodiversity will inevitably impair vital services provided by nature. In the Millennium Ecosystem Assessment report [16], ecosystem services are defined as “the benefits people obtain from ecosystems.” They are divided into four interrelated categories: provisioning (e.g., food, wood), regulating (e.g., climate, flood), cultural (e.g., aesthetic, recreational), and supporting (e.g., nutrient cycling, soil formation). With loss in biodiversity, not only will some of these services be directly taken away, the ones that still remain will also become less resilient. In the context of ecosystem services, resilience is defined as the “degree to which an ecosystem function can resist or recover rapidly from environmental perturbations, thereby maintaining function above a socially acceptable level [17].” When the number of species decreases in an ecosystem, resilience is reduced through the losses of both redundancy and interconnections of the components in the system [17], hence affecting its ability to bounce back in environmental disturbances. The deteriorated ecosystem will inevitably affect the services it provides.

The ecosystem resilience is intertwined with societal resilience. Sometimes, an acceptable level of ecosystem service at normal time may become inadequate when a society faces stresses. For example, with urban sprawl, rural areas near cities are replaced by infrastructures, and water bodies are heavily polluted by urban stormwater runoffs. The original ecosystem is severely degraded along with the provisioning services. With well-developed transport systems, a city typically imports most of its fresh produce from remote rural regions. Hence, demands for farming products from its suburban area become minimal. However, with some severe disturbances such as the current Covid-19 pandemic, the transport systems may be severely disrupted due to lockdowns, causing the shortage and price increase of fresh produce. Supplies from local farmers are no longer able to fill the gap due to the loss of arable land, clean water for irrigation, and pollinators. The financially disadvantaged people will suffer most from the price increase of produce. Therefore, maintenance of a healthy ecosystem is beneficial for cities even from the aspect of “provisioning,” not to mention other services provided by nature to urban dwellers such as clean air and water.

This paper addresses the influences of civil infrastructures on biodiversity. More specifically, it is focused on the impacts of linear infrastructures such as highways or railways on pollinator insects (pollinators hereafter) in suburban areas and possible ways to improve infrastructure planning and design to enhance pollinator biodiversity. Pollinators provide important ecological services. A study by Klein et al. [14] found that 87 of the important global food crops depend on pollinators, while 28 crops do not. Yet, pollinators are declining globally at an alarming rate, accompanied with the declines of plants that rely on the pollinators [19]. Causes behind the losses in pollinators can be mostly attributed to anthrogenetic activities, including habitat fragmentation and loss, chemical uses in agriculture, pathogens, alien species, climate change and interactions of these factors [19]. The negative impacts of linear infrastructures are manifested in habitat segmentation and loss, road kills, and perhaps air pollution from vehicles. For instance, Andersson et al. [1] conducted a study on the species composition of bees and wasps on two sides of a large highway in Sweden and found there was a significant difference in species composition between the two sides of the road. The findings proved that roads segment the populations of flying insects. Fuentes et al. [8] found that even moderate amount of atmospheric pollutants such as ozone, nitrate radical, and hydroxyl radical may cause rapid degradation of floral scents, which help pollinators find food and help plants attract pollinators to complete the pollination process.

In suburban metropolitan areas, even if some natural lands are preserved, they are often heavily segmented by highways or railways. Out of the possible impacts of linear infrastructures on pollinators, this paper is focused on their effects on flying insect pollinators’ foraging activities. Habitat connectivity is very important for the resilience of inset pollinators. Prior to the construction of the infrastructures, the pollinators can move freely in connected lands, foraging in a large area for food supplies. After the infrastructures are built, the movement of pollinators is constrained. In particular, flying across the highway not only becomes dangerous, it is also not lucrative as the pollinators may not detect strong floral scents from the other side of the highway.

Scents play an essential role in pollinators’ foraging activities [20]. In a natural environment, certain flowers produce scents in the form of volatile organic compounds (VOCs) to attract pollinators. Pollinators such as bees pick up the scents and try to find their ways to locate the source of scents. This may be done by following the gradient of scent emitted from the source, with the assistance of visual images. Once the source is identified, in the case of bees, the foraging bee will return to the hive and perform the well-known “dance” to inform co-workers on the location of the source. It is believed that the locational information conveyed by “dance” is not very precise [20]. Once the recruited bees fly into the general area of flower source, they need to identify the exact location by either visual images or scents [20]. If the flower source covers a large area, it would be easy to identify the source visually. However, when the source is small and isolated, which is typical for flowers in urban and suburban areas, scents become more important. The intensity of flower scents is correlated with the distance and amount of food reward—nectar or pollen [6]. In addition, foraging honey bees also release some pheromones such as Nasonov pheromone, in which some compounds are similar to those in floral scent, to attract others to specific locations such as food source [7]. Moreover, some bee species such as stingless bees do not use “dance” to communicate the flower location at all; instead, they leave a trail of odor between the nest and the flower site to guide nestmates to find the food source [20]. As shown in the summary from the paper by Reinhard and Srinivasan [20], a corridor of scents or odors is critically important for pollinators such as bees to find food, which in turn help cross-pollinate plants. The process benefits both the abundance and diversity of the pollinators as well as the plants.

However, the presence of linear infrastructures such as highways forms a man-made barrier by increasing the distance between nests and food sources. The increased distance not only dilutes the scents emitted from a flower source across highways but also makes it impossible for some bee species to leave a continuous odor trail. Air turbulence from the vehicles and air pollutants from vehicle emissions and asphalt pavements may also become additional stressors. Emissions from vehicles may react with scents and alter their chemical compositions [8]. Both processes may be detrimental to flying pollinators and some plants to be pollinated.

The overall goal of this study is to evaluate the influences of atmospheric environment near highways on the dispersion of floral scents and the effectiveness of using green corridors across highways to enhance the connectivity of habitats in floral scent dispersion. The study includes the following specific objectives: (1) to understand differences in air between a natural environment and a roadside environment and the implications of such differences on floral scents, (2) to understand the influences of different green corridor configurations on floral scent dispersion. It is anticipated that the study will encourage investigating the possibility of retrofitting traditional infrastructures to improve the connectivity of pollinators in different habitats, thereby enhancing the resilience of ecosystems and biodiversity.

## Research method

This research is performed in the context of Hong Kong, a major city located on China’s southern coast. Although Hong Kong is among the most densely populated places, about 70% of the total territory is undeveloped green areas, and 40% of the land area is officially designated to parks [10]. The geographical location, mild climate, and ample green areas of Hong Kong nurture rich biodiversity that is quite unique for a large city. For example, it hosts more than 3300 species of vascular plants including 2100 native ones, more than 540 species of birds, 86 species of reptiles, 24 species of amphibians, 236 species of butterflies, and 123 species of dragonflies [11]. The green areas of Hong Kong, however, are often segmented by linear infrastructures as well as dense buildings. In fact, with rapid urbanization, the entire Pearl River Delta region in China’s southern coast is heavily segmented, where limited natural lands are separated by built infrastructures. To improve the resilience of wild lives, the isolated natural lands need to be re-connected using different strategies. Cross-highway green corridors are selected as a possible type of green infrastructure for the purpose of improving the connectivity of pollinators.

### Study area and an assumed case

**Fig. 1:**
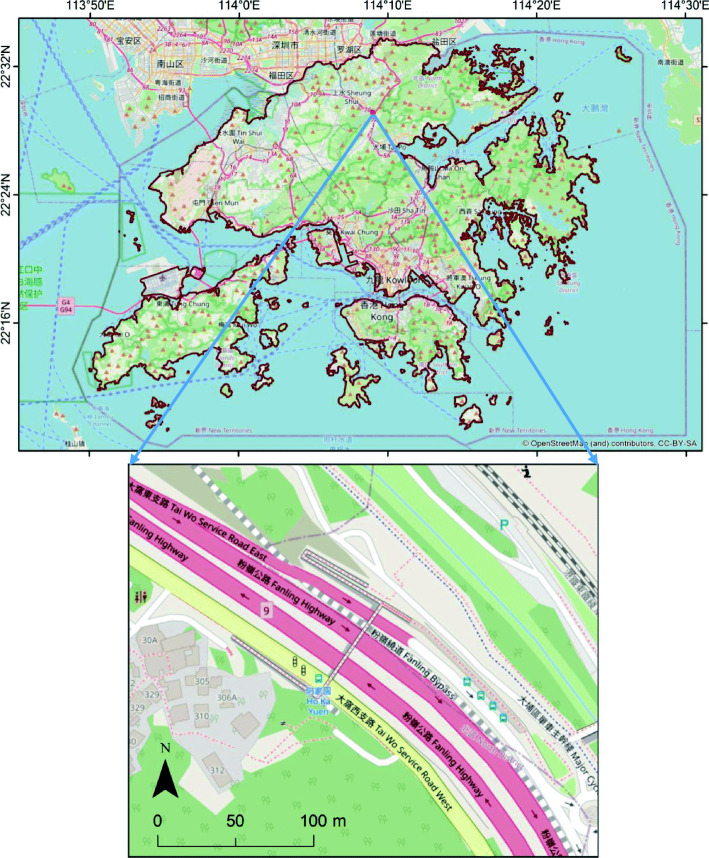
Location and map of the assumed case

The following methods are used to assist the investigation. Firstly, an area of 300 m × 250 m including a section of a major highway (Fanling Highway) in the North District of Hong Kong is selected for micro-scale study (Fig. 1). Currently, there are several overpasses along the highway. This study evaluates the effect of converting an overpass to a “green corridor,” by planting flowers on the covers of the overpass to disperse floral scents and attract pollinators to cross the road. As shown in the Fig. 1, large green areas are separated by the highway and a nearby railway, along with built-ups which are mostly residential buildings. The green patches on the left consist of a small orchard and isolated fruit trees. They are connected to mountain areas that are relatively dry because moist air from the east is blocked, hence the landscape is dominated by drought-resistant grass and short shrubs. In particular, rose myrtle (*Rhodomyrtus tomentosa*) and common melastoma (*Melastoma malabathricum* L.) are two common species, which produce flowers from late spring to early fall, attracting a variety of pollinators that in turn benefit the shrubs by promoting the production of fruits. The fruits are important food sources for birds. The mountain areas on the right are relatively wet and hence are dominated by trees, including commonly seen ivy trees (*Schefflera octophylla*) which bloom from late fall into winter. There are also small orchards in the region. Together, the diverse landscape provides complimentary food sources and nesting places for pollinators all seasons. This section of highway is used to evaluate how flower scents from one side of the highway and railway are affected with different configurations of a pedestrian footbridge over the highway.

**Fig. 2:**
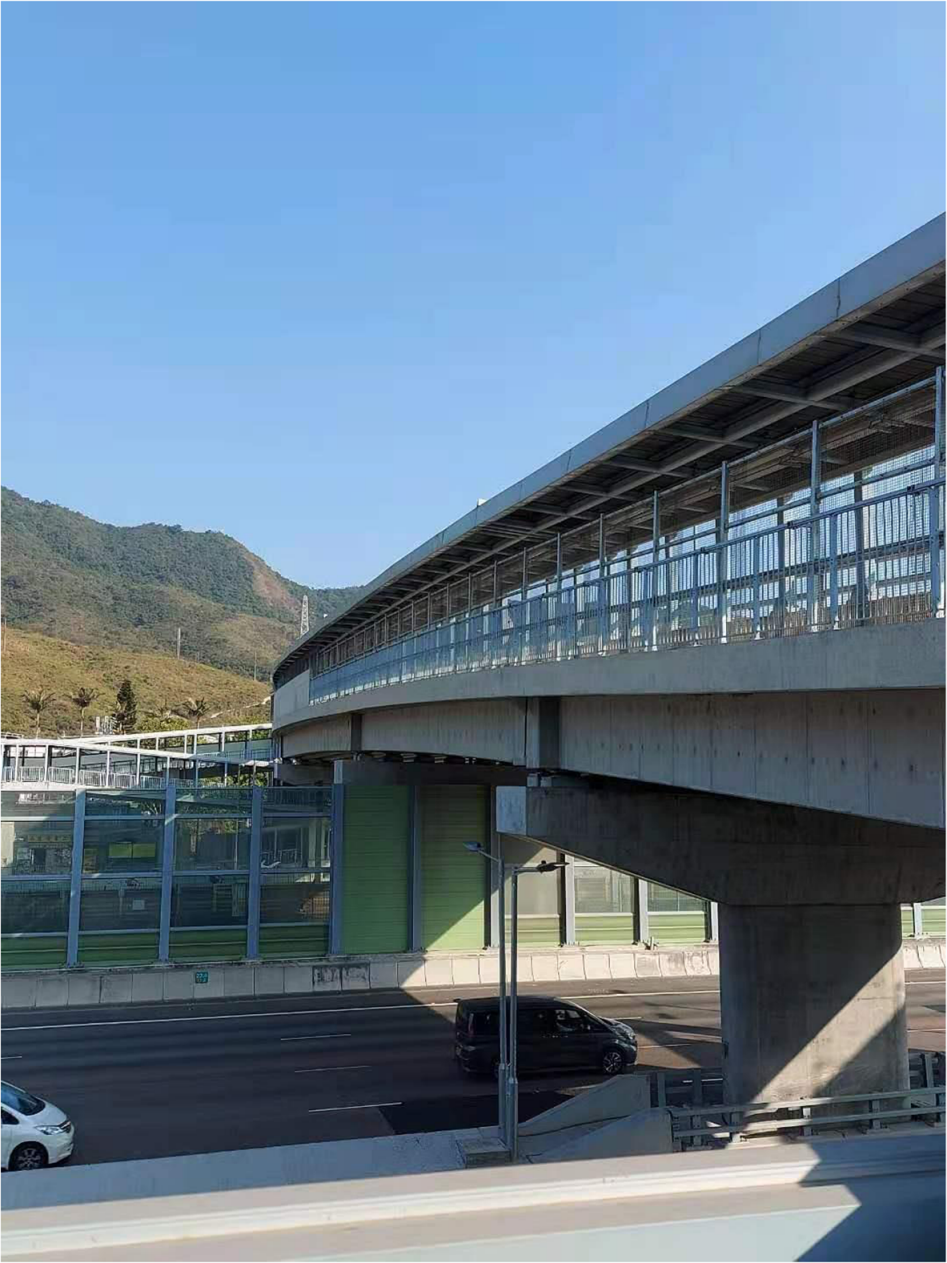
The current design of highway overpass for pedestrian traffic

An actual photo of the highway overpass for pedestrian traffic is shown in Fig. 2. Also can be seen in the photo is the natural environment close to the highway. In Hong Kong, these footbridges typically have a metal cover with a robust structure design. Little difficulty would be encountered to retrofit the cover by introducing flowering plants onto the cover. Moreover, barriers may be installed on the cover to reduce air turbulence and traffic noise, confine the floral scent, and reduce the likelihood of road kills.

### Comparisons of natural and roadside atmospheric environment

Some of the air quality monitoring data is provided by the Environmental Protection Department (EPD) in Hong Kong [12]. The monitoring network consists of 18 stations, including 3 roadside ones. The monitored air quality parameters include carbon monoxide (CO), fine suspended particulates (FSP), nitrogen dioxide (NO_2_), nitrogen oxides (NOx), ozone (O_3_), respirable suspended particulates (RSP), and sulphur dioxide (SO_2_). Hourly monitoring data is available for these stations. Two of the stations—Tap Mun monitoring station and Mong Kok monitoring station—are selected to understand differences in air parameters. The former is located in a remote island in Hong Kong without automobiles, and the sampling location is 11 m above the ground. Hence, the recorded air parameters represent air conditions in a natural environment. The latter is located near an urban road with buildings along the two sides of the road, used to represent typical air parameters in an urban street canyon [12]. The monitoring data from March 28 to May 32,015 are selected and summarized in this paper. This time period is chosen because an air monitoring experiment studying the photochemical oxidation processes in urban street occurred during the same period of time [23]. The estimation of another two essential atmospheric oxidants, hydroxyl radical (OH) and nitrate radical (NO_3_), were based on this experiment.

It has been very challenging to directly measure the hydroxyl radical (OH) and nitrate radical (NO_3_). The OH is the most important photochemical oxidant in the atmosphere and plays a central role in air quality. Most of the OH is produced from the reaction of water vapor with O(^1^D) produced from the photolysis of O_3_. Therefore, it shows a clear diurnal pattern with peak concentration at noon and near-zero concentration at night. The nitrate radical (NO_3_) is formed from the reaction of NO_2_ and O_3_. During the daytime, it will rapidly photolyze at a rate of 0.3 s^− 1^, and therefore its concentration can only accumulate during nighttime. At night, NO_3_ can react with NO to produce NO_2_, or further react with NO_2_ to produce dinitrogen pentoxide N_2_O_5_. The N_2_O_5_ can decompose back to the two reactants thermally, thus forming a fast dynamic NO_3_-N_2_O_5_ equilibrium [21]. Their concentrations were estimated from previous observation data measured at a roadside monitoring station (Mong Kok) and a remote background supersite (Hok Tsui). Like the Tap Mun site, Hok Tsui is deemed as a natural site that is less affected by urban emissions. The OH concentration was estimated using atmospheric photochemical models with the input of measured trace gas data. Details on measurements, modeling and estimation methods can be found in the literature [22, 23].

As mentioned before, floral scents play a key role in helping pollinators find food sources. Among them, there are 12 odorants that occur in over 50% of all floral bouquets analyzed and are hence regarded as typical floral odorants: limonene, (E)-b-ocimene, myrcene, linalool, a-pinene, b-pinene, benzaldehyde, methyl salicylate, benzyl alcohol, 2-phenyl ethanol, caryophyllene, and 6-methyl-5-hepten-2-one [20]. Fuentes et al. [8] performed a simulation on the floral scent degradation under atmospheric oxidants (O_3_, OH, and NO_3_) using 5 of the 12 species (b-caryophyllene, b-ocimene, b-myrcene, linalool, and a-pinene). They used Large Eddy Simulations (LES) to simulate the spatial and temporal variations of floral scents due to oxidation. Changes in composition and concentration of the floral scents with varying content of the three oxidants were also estimated. Based on the same concept, floral scent degradation rate at a natural site and roadside site is compared based on the measured O_3_ levels and estimated OH and NO_3_ levels in the two environments:

1 $$ {\delta}_i=\frac{k_{O_3}(i)\ast {\tilde{x}}_i(natural)\ast {O}_3(natural)+{k}_{OH}(i)\ast {\tilde{x}}_i(natural)\ast OH(natural)+{k}_{NO_3}(i)\ast {\tilde{x}}_i(natural)\ast {NO}_3(natural)}{k_{O_3}(i)\ast {x}_i\left(r\tilde{o} ad\right)\ast {O}_3(road)+{k}_{OH}(i)\ast {\tilde{x}}_i\ast OH(road)+{k}_{NO_3}(i)\ast {\tilde{x}}_i\ast {NO}_3(road)} $$

Where ${k}_{O_3}(i)$, *k*_*OH*_(*i*), ${k}_{NO_3}(i)$ are the reaction rate coefficient [2] of O_3_, OH, and NO_3_ for a particular floral VOC *i*;

${\tilde{x}}_i(natural)$ is the floral VOC *i* concentration in a natural environment while ${x}_i\left(r\tilde{o} ad\right)$ is the VOC *i* concentration in a roadside environment;

*O*_3_(*natural*), *OH*(*natural*), *NO*_3_(*natural*) is the O_3_, OH, and NO_3_ concentration in a natural environment, respectively, and similar notations are used for the roadside site.

Assume that the floral VOC concentration is the same at the two site, the ratio of degradation rate becomes:

2 $$ {\delta}_i=\frac{k_{O_3}\ast {O}_3(natural)+{k}_{OH}\ast OH(natural)+{k}_{NO_3}\ast {NO}_3(natural)}{k_{O_3}\ast {O}_3(roadside)+{k}_{OH}\ast OH(roadside)+{k}_{NO_3}\ast {NO}_3(roadside)} $$

This ratio is used to approximately evaluate the impacts of natural atmospheric environment and roadside environment on floral VOC degradation.

**Fig. 3:**
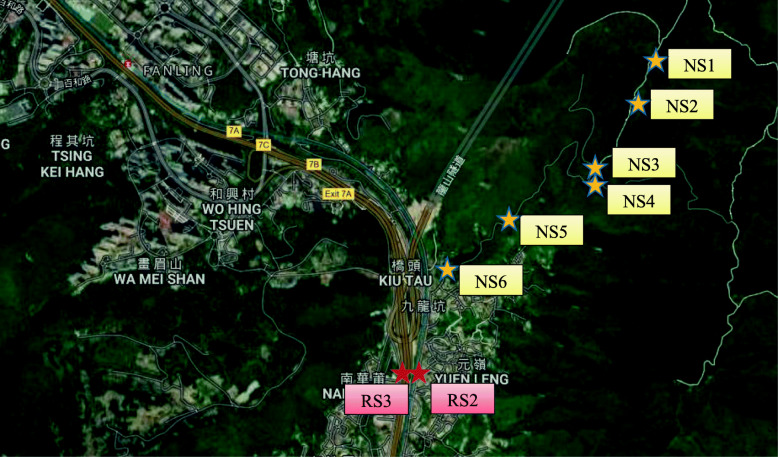
Locations of field air parameter measurements

To better understand differences in air parameters between the natural environment and the roadside environment, a portable gas analyzer (GASMET DX4040) was used to measure air parameters at eight sites near the case study area (Fig. 3). The measurement was taken on a sunny day with low wind. RS2 and RS3 are located on a footbridge above the major highway and on the top edge of the noise barrier of the highway, respectively. Air samples obtained at these two locations represent air over the highway. NS1 to NS3 are located at the east side of the mountain, NS4 is located at the ridge of the mountain, and NS5 and NS6 are located at the west side of the mountain. As compared to NS1 to NS3, NS5 and NS6 are likely more affected by the built-up area. The gas analyzer provides a total of 45 air parameters, most of which are VOCs. To make the results more comparable, the measurements took place in a short time window starting at 14:04 and ending at 15:47. The measured value at the mountain ridge (NS4) is used as a reference, based on which the relative air parameters are calculated. Due to the lack of cross-validation of the measured data with more precise laboratory air analyzers, the measured data only serve as indicative purpose, not for quantification.

### Simulations of floral scent dispersion over green corridors

A micro-scale CFD model—ENVI-met—was used for its capability to simulate diverse planetary boundary layer processes such as wind flow, turbulence, microclimate, and pollutant dispersion [4]. In this model, the dispersion of particles or gas is calculated using the standard advection-diffusion equation. In this study, one of the typical species of floral VOC - *β*-ocimene is used to estimate its dispersion patterns. The emission rate of the targeted VOC type is 82 nmoles m^− 2^ min^− 1^ [8]. Key input parameters for ENVI-met include weather conditions, initial soil wetness and temperature profiles, structures and the physical properties of urban surfaces, as well as plants [3].

The model space is digitized into a horizontal resolution of 5 m and a vertical resolution of 2 m. Four types of land cover are considered for the study area: soil, concrete pavement, woodland, and shrubland. The buildings (wall albedo: 0.25, roof albedo: 0.3, height: 30–60 m), trees (plant albedo: 0.2, height: 10 m) and shrubs (plant albedo: 0.2, height: 0.5 m) are distributed on those land-cover types. An overpass (height: 10 m) is located above the highway.

**Fig. 4:**
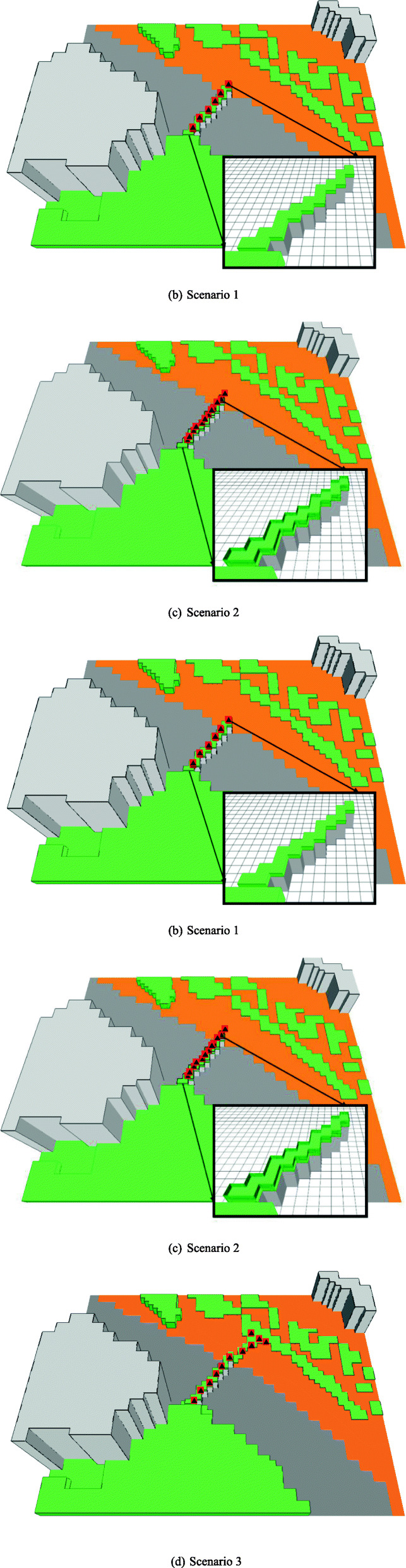
The layouts of simuation scenarios

Different simulation scenarios are assumed. In the base scenario (Fig. 4a), the overpass is not covered with a flower bed, and 50% of a green strip at the simulated area’s upper-right corner is covered by flowers. The flowers are assumed to emit a VOC *β*-ocimene at a rate of 82 nmoles m^− 2^ min^− 1^ [8]. Three other simulation scenarios are developed to evaluate the influences of different green corridor configurations on VOC dispersion. In the first simulation scenario (Fig. 4b), a green corridor made of flower bed (length: 100 m, width: 50 m, height: 0.5 m) is applied onto the current existing overpass. In the second scenario (Fig. 4c), two barriers (height: 2 m, length: 100 m for each side) are installed along the green corridor to block the dispersion of floral scents from the traffic directions. In the third scenario (Fig. 4d), the green corridor is the same as that in scenario one, except for that the greening area is extended to the surrounding areas with a total length of 160 m.

**Fig. 5:**
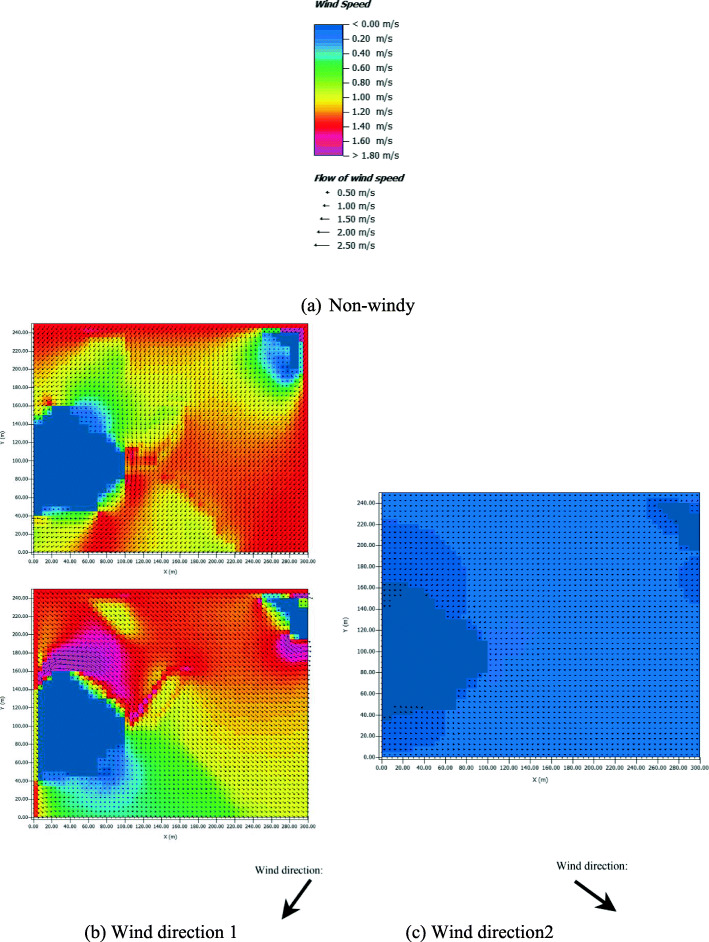
The distributions of wind speed and direction in the simulation area under the three wind conditions

Simulations are based on an assumed mild temperature range (18 − 24 ° *C*). Three wind conditions are considered: non-windy and two windy conditions with different directions (Fig. 5). Due to the model limitation of ENVI-met, the minimum wind speed that can be set is 0.1 m/s. The wind direction of the first windy condition is perpendicular to the road (36.87° south), while the wind direction of the second windy condition is parallel to the road (306.87° south). Simulation details are summarized in Table 1.

**Table 1:** The simulation details

Size of simulation area	300 m × 250 m
Size of grid cells (*dx*, *dy*, *dz*)	5 m × 5 m × 2 m
Simulation period	08:00–17:00
Air temperature	18 − 24 ° *C*
Wind speed	0.1 m/s
Wind direction	North
Relative humidity	50 − 70°

## Results and discussion

### Comparions of air parameters

**Fig. 6:**
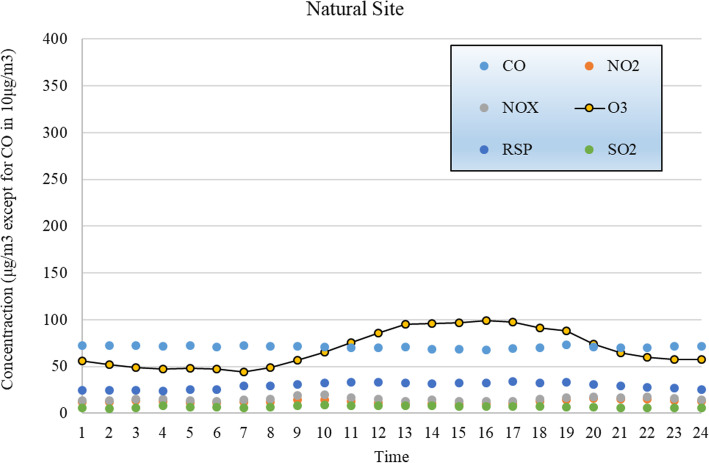
Air quality parameters at a natural site (Tap Mun, Hong Kong)

**Fig. 7:**
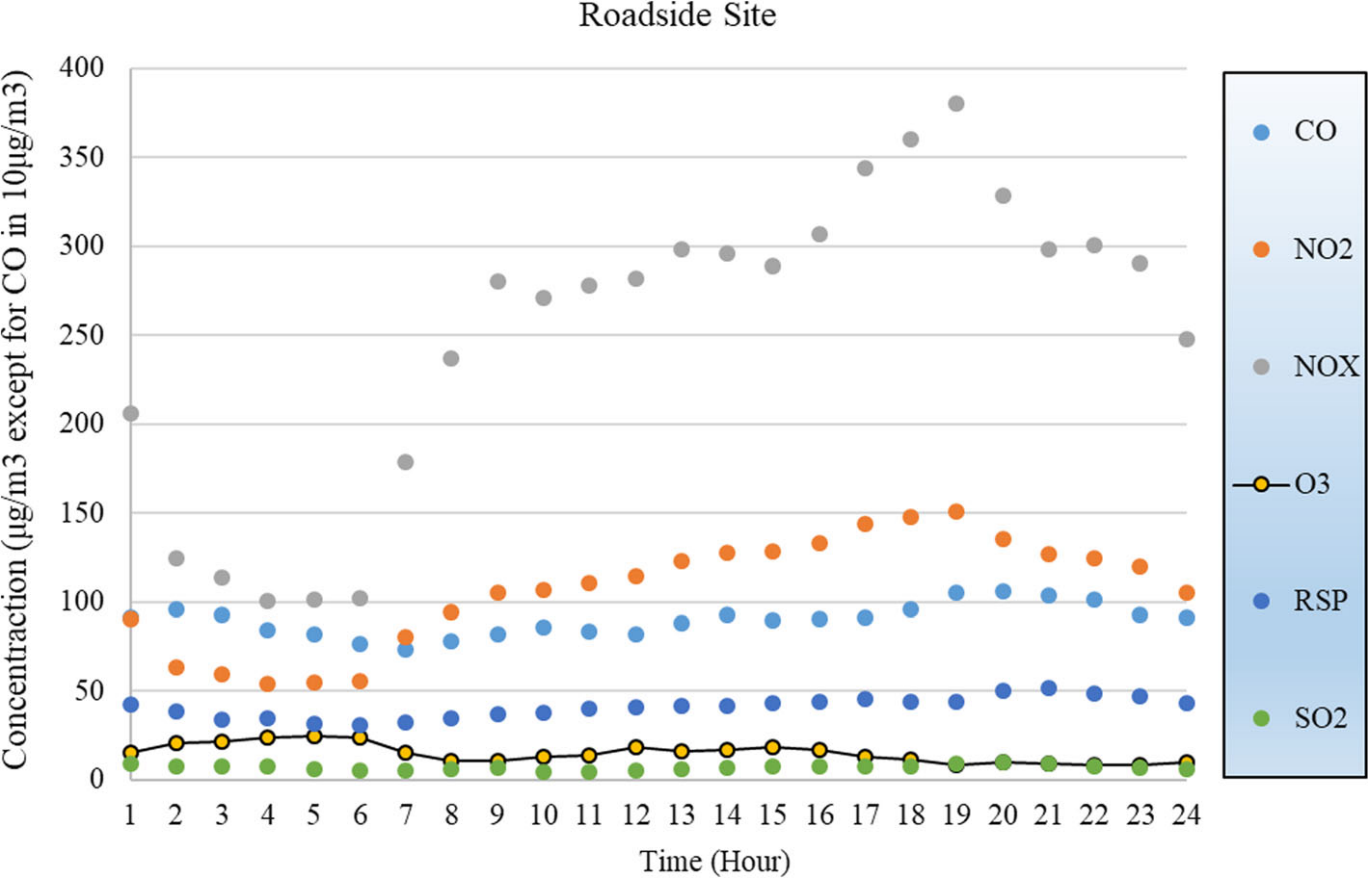
Air quality parameters at a roadside site (Mong Kok, Hong Kong)

The average hourly concentrations of the air quality data from the two monitoring stations during the selected period are shown in Figs. 6 and 7. As shown in the figures, the level of RSP at the natural site is lower than that at the roadside site, and the levels of SO_2_ at the two sites are similar (average 6.92 μg/m^3^ at the Tap Mun site vs. average 7.07 μg/m^3^ at the Mong Kok). Although the level of CO is also similar, that in urban roadside shows more fluctuation. The levels of NO_2_, NOx (NO+NO_2_), and O_3_, however, differ dramatically. In particular, the natural site shows a much higher level of O_3_ while very low levels of NO_2_ and NOx. The abundance of NO_2_, NO_x_ and VOCs generated from vehicles in urban roads causes the reduction in O_3_.

The OH concentration was estimated using atmospheric photochemical models with the input of measured trace gas data. The calculated peak concentration was around 0.26 pptv at the natural site (Hok Tsui) and 0.18 pptv at the roadside site (Mong Kok) [22, 23]. The NO_3_ concentration was estimated from the N_2_O_5_ measurement conducted at the two aforementioned sites, with peak concentrations of 6.2 and 1.4 pptv at the natural site and urban roadside during the night, respectively [22, 23]. In the daytime, NO_3_ concentrations at both sites are close to zero. Using the monitored O_3_ concentrations and calculated OH concentrations at the natural site and roadside site, the degradation ratios of the five floral VOCs are calculated and shown in Table 2. The degradation ratios indicate that the chosen floral VOCs are more easily degraded in a natural environment than in a roadside environment.

**Table 2:** The calculated degradation ratios (nature/roadside) of the five floral VOCs

VOC Type	Reaction Rate			Degradation Ratio
	K_O3_(cm^3^ molec^−1^ s^− 1^)	K_OH_ (cm^3^ molec^− 1^ s^− 1^)	K_NO3_(cm^3^ molec^− 1^ s^− 1^)	
b-Caryophyllene	1.1E-14	2E-10	1.9E-11	4.50
b-Ocimene	5.4E-16	2.52E-10	2.2E-11	1.77
b-Myrcene	4.7E-16	2.13E-10	1.27E-11	1.78
Linalool	4.3E-16	1.59E-10	1.12E-11	1.86
a-Pinene	8.09E-17	5.33E-11	6.16E-12	1.66

With reference to the point at mountain ridge (NS4), data measured by the portable air analyzer (GASMET DX4040) are compared, with focus being placed on VOCs. The following chemicals are found to be higher at the west side of the mountain than at the east side, and the differences are above the detection limits: toluene, acetic acid, diethyl ether, chloroform, dimethylamine, and m-Xylene. The only VOC that is slightly higher at the east side of the mountain is ethyl acetate. The better air quality at th east side of the mountain is understandable as it is seperated from the built-up areas by the mountain (Fig. 3). As compared with the east side of the mountain, the following chemicals are found to be higher above the highways: benzene, toluene, acetic acid, diethyl ether, ethylene oxide, methyl mercaptan, chloroform, dimethylamine, m-Xylene, ethane, and Ethylene. Ethyl acetate and fluorobenzene over the highway are slightly lower. The results indicate that air above the highway contains higher concentrations of VOCs as compared to air in a more natural environment. Some of the higher concentrations of VOCs are likely related to automobiles and asphalt pavements.

In summary, floral scents above highways would degrade slower as compared to a natural environment if flowers are planted over a highway overpass, due to the lower concentrations of oxidative radicals in the air. Emission from vehicles generate chemicals that react with the atmospheric oxidants. Conversely, highway air contains higher amount of other types of VOC species that are likely related to emissions from automobiles and pavements.

### The floral VOC dispersion of different designs of green corridors

The foral VOC dispersions in the daytime (from 08:00 to 19:00) from the three scenarios are generated by ENVI-met. The foral concentration is found to peak at about 14:00, which is selected as the time point to present the concentration results in the following figures. In addition, VOC concentrations above the highway overpass are compared.

**Fig. 8:**
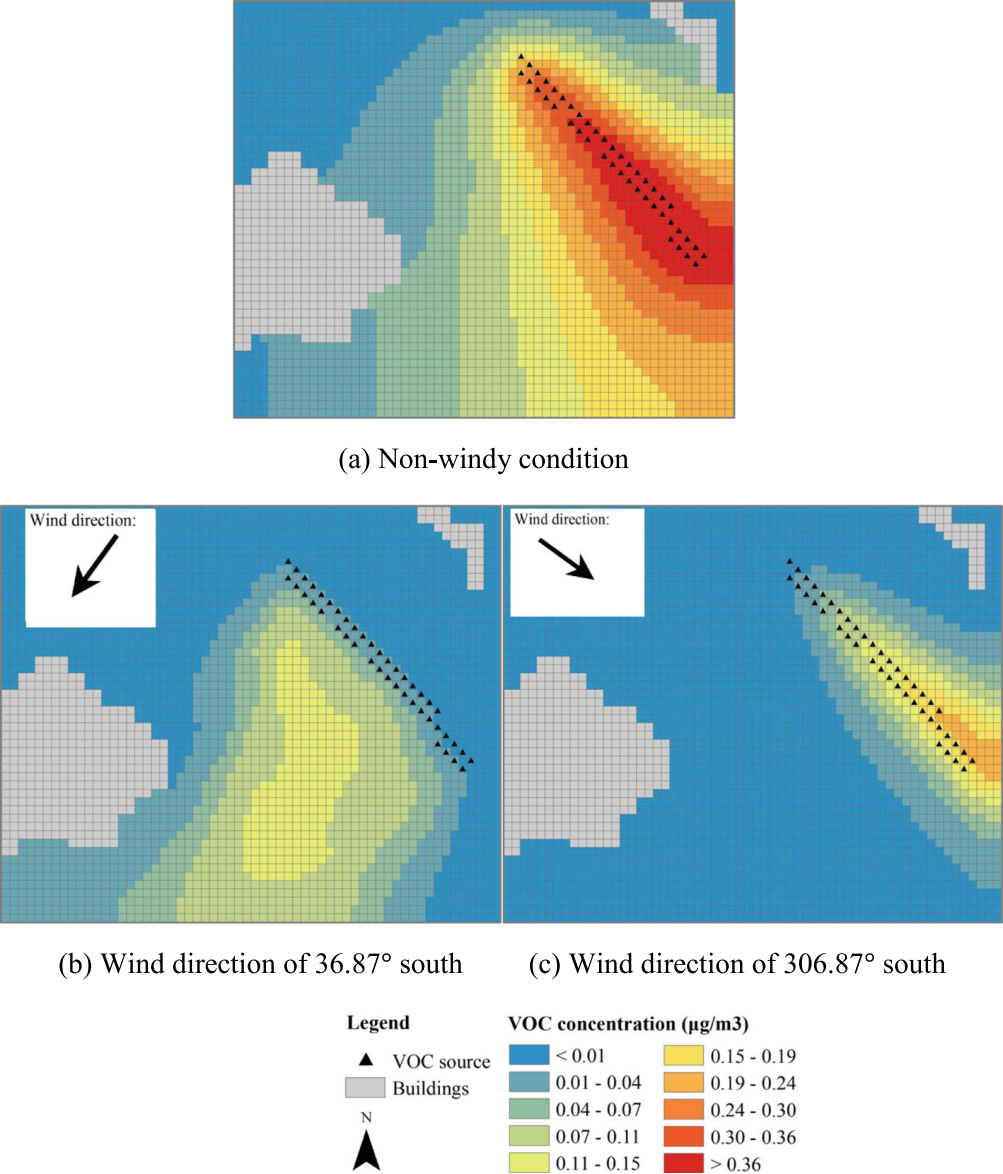
VOC concentrations of the base scenario

The VOC concentrations of the base scenario are shown in Fig. 8. Note that in this scenario the flowers are sparsely located in a green strip at the right side of the road. In a non-windy condition, the VOC concentration directly above or near the flowers is the most intensive. The concentration decays rapidly with distance. The concentration across the road is only about 1/5 to 1/10 of that nearly the flowers. In windy conditions, the locations with the highest concentration of VOC are shifted from the location of the flowers. When the wind direction is perpendidular to the highway, the intensity of VOC across the road is similar to that of non-windy condition. When the wind direction is parallel to the highway, however, no floral VOC is dispersed across the road. The highway indeed acts as a physical barrier for floral scent.

**Fig. 9:**
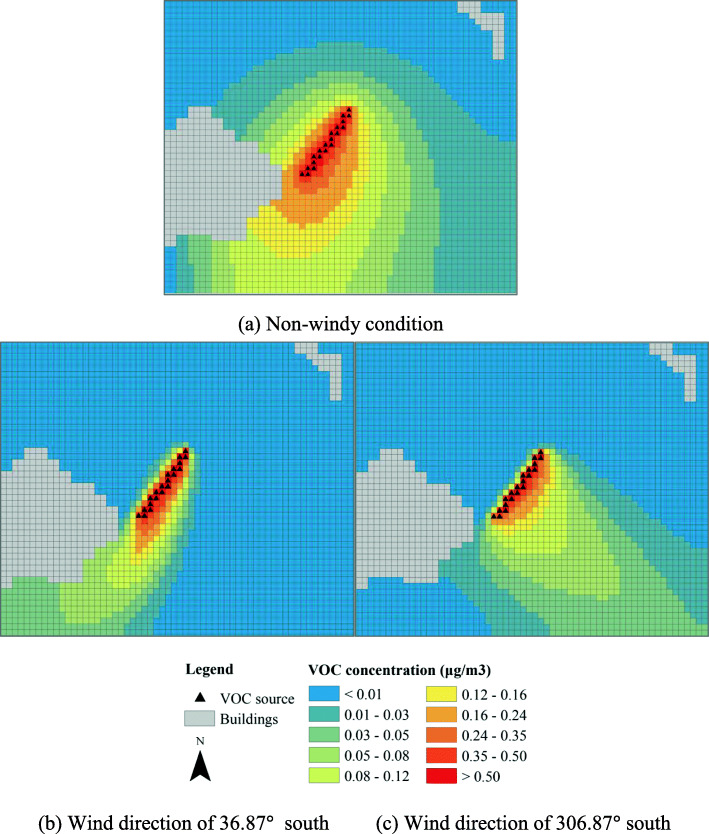
VOC concentrations of an open green corridor

Figure 9 shows the dispersions of the VOC after the creation of an open green corridor that is planted with a flower bed. Note that emission from the on-ground green strip in Fig. 8 is not included in the simulation, and the intensity scale represented by different colors are also different in Figs. 8 and 9. As shown in Fig. 9, high intensity of the VOC can be found on the corridor as well as at the left side of the road, even when the wind condition is unfavorable (perpendicular to the road). The open green corridor with flower bed may effectively serve as a bridge for pollinators.

**Fig. 10:**
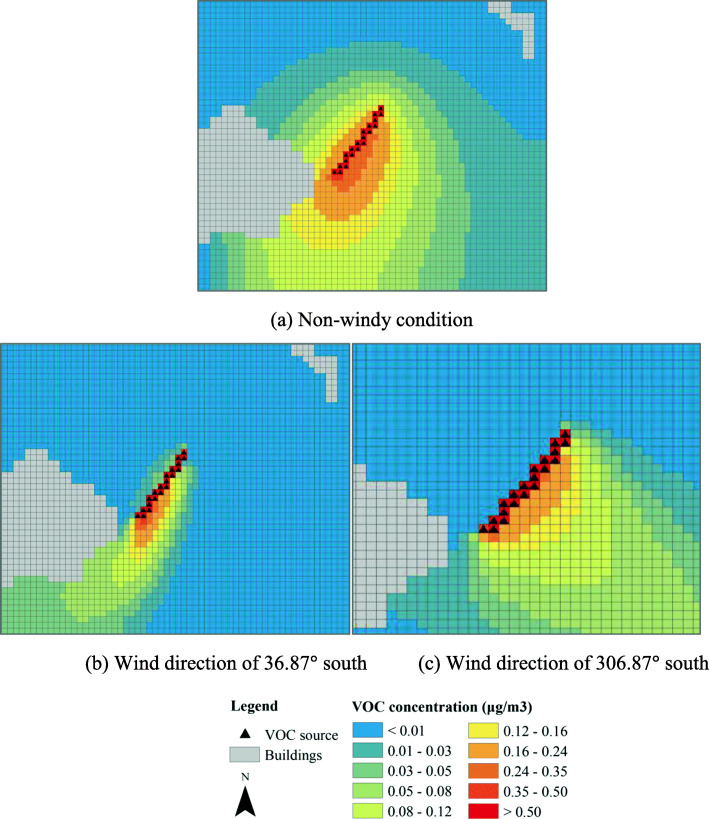
VOC concentrations of a semi-closed green corridor

Figure 10 shows the dispersions of the VOC with a semi-closed flower bed at two sides by a barrier of 2 m high. The intensity distributions under the three wind scenarios are similar to those in Fig. 9, especially for the edge of the regions covered by the dispersed VOC. However, the regions of high-intensity VOC at and near the flower bed are narrowed, indicating that the VOC is more concentrated within the semi-closed flower bed. In general, either open or semi-closed flower bed does not largely influence the dispersion of the floral VOC.

**Fig. 11:**
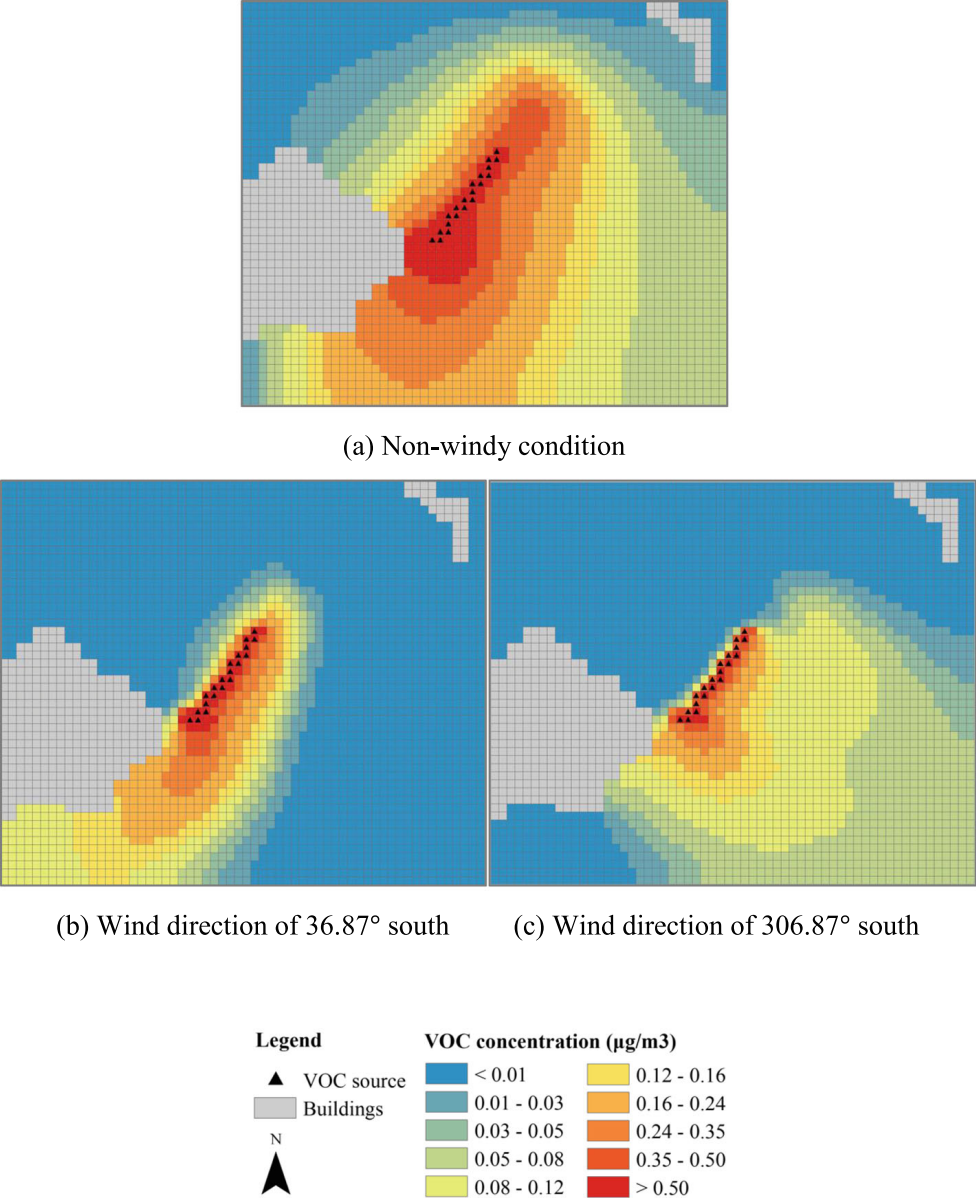
The VOC concentration of the green corridor with additional flowers planted at the right end of the bridge

Figure 11 shows the dispersions of the VOC in scenario 4. In this scenario, flower plants are also created at the right end of the bridge to connect to the green strip. As shown in Fig. 11a, in a non-windy condition, region with high intensity of the VOC is much expanded as compared to those in Figs. 9 and 10. Even in unfavorable wind, high intensity of VOC can be seen at the left side of the bridge. The results suggest that the creation of the connector flower plants can significantly improve the dispersion of the VOC in all conditions.

### Consideration of plants for the green corridors

The simulation results clearly indicate the benefits of planting flower beds on and near the highway overpass. Besides engineering design, another important consideration is the selection of plant species for the green corridors. Plants chosen on the bridge cover may be short and request low maintenance, while plants on the ground may be tall to provide better connection with those on the bridge. Plants need to be selected to attract native inset species, especially those endangered or charismatic ones. In addition, a variety of plants may need to be carefully selected to provide food for different pollinators in different seasons. Bees are the primary pollinators, and have attracted much discussion due to their close relationships with the flowering plants [13] and the high contribution to fruit-pollination services [18]. Bees are disappearing at an alarming rate due to habitat loss [15] and other causes. Up to now, bees in Hong Kong have not attracted enough attention for protection. Butterflies also play critical roles in ecosystem services. For example, at least six plants rely on butterflies to disperse pollens in Hong Kong [9]. In total, there are 245 species of butterflies covering five families that inhabit Hong Kong, including some endemic ones such as *Halpe paupera walthewi* [9]. Although Hong Kong has 13 designated sites strengthening butterfly conservation, improving connectivities of different habitats would increase the resilience of butterfly abundance and diversity. As butterflies have their own specific host plants, such plants may be included in the “portofolio” of plants in the green corridors.

**Fig. 12:**
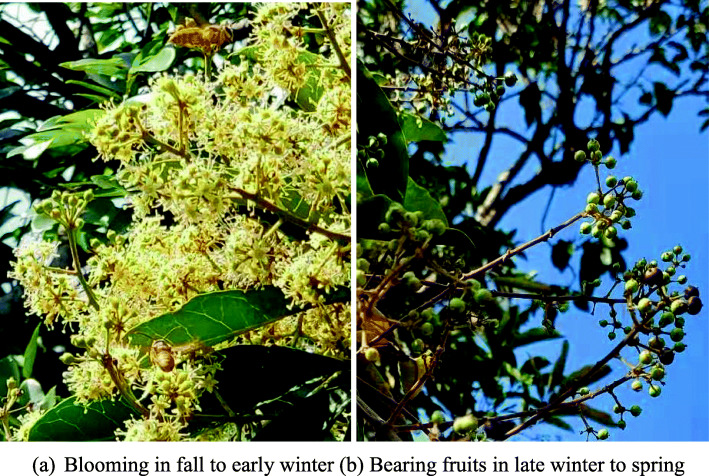
An example of native tree (*Schefflera heptaphylla)* that provide rich ecological services to local insect pollinators and birds

Moreover, native plant species provide habitats for local birds and insects, thus enhancing the biodiversity and ecological succession of the city. For example, the ivy tree (*Schefflera heptaphylla)* (Fig. 12) as introduced before is a native evergreen tree in Hong Kong, which is a valuable tree for various animals. The white flowers are one of the main nectar sources for bees and other insects in the local winter. It is also the host plant for butterfly family *Hesperiidae’s* larvae. In addition, the berries of the plant are favorite foods for forest birds such as the *Zosterops japonica.* Hence, the plant may be chosen as one of the roadside connector plants.

## Summary and conclusions

Biodiversity conservation remains a grand challenge globally. In a highly compacted city like Hong Kong, there is a continuous demand of land for housing, quality public space, infrastructure and facilities [11]. To reach the aim of sustainable development together with biodiversity conservation, innovative techniques are needed to incorporate biodiversity into urban planning and infrastructure design.

This paper discusses the possibility of introducing green corridors on highway overpass to increase the connectivity of habitats for flying pollinator insects. The physical and chemical environment of highways poses great challenges for pollinators to cross the road. It is found that if flower plants are installed on highways, the floral scents emitted by the plants are actually better preserved as compared with those in a natural environment due to the lower concentrations of oxidative radicals that react with the floral scents. As a result, the floral scents over highways may be stronger and be transported further. This is a potential advantage of growing floral plants over highways. Conversely, highway operations and materials emit more VOCs as compared with the natural environment. The effects of different VOC compositions on pollinator insects’ foraging behaviors are currently unknown. Highways indeed form a physical barrier for floral scent to disperse from one side to the other. The use of green corridors on the highway overpass can greatly improve the connectivity of floral scents, especially when on-ground flowering plants are also introduced near the highway. The use of a properly selected combination of plant species and properly designed engineering features is believed to help improve the habitat connectivity of pollinator insects, thus improving the resilience of declining pollinator biodiversity. The effectiveness of such practice, however, still needs to be proved through engineering practices.

## Data Availability

The datasets used and/or analysed during the current study are available from the corresponding author on reasonable request.

## Abbreviations

CFD: Computational fluid dynamics
VOC: Volatile organic compound
EPD: Environmental Protection Department
CO: Carbon monoxide
FSP: Fine suspended particulates
NO_2_: Nitrogen dioxide
NOx: Nitrogen oxides
O_3_: Ozone
RSP: Respirable suspended particulates
SO_2_: Sulphur dioxide
OH: Hydroxyl radical
NO_3_: Nitrate radical
N_2_O_5_: Dinitrogen pentoxide
LES: Large Eddy Simulations

## References

[1] Andersson P, Koffman A, Sjödin NE, Johansson V (2017) Roads may act as barriersto flying insects: species composition of bees and wasps differs on two sides of a large highway. Nat Conserv 18:47–59. 10.3897/natureconservation.18.12314

[2] Atkinson R, Baulch DL, Cox RA, Hampson RF Jr, Kerr JA, Rossi MJ, Troe J (1999) Evaluated kinetic and photochemical data for atmospheric chemistry, organic species: supplement VII. J Phys Chem Ref Data 28(2):191–393. 10.1063/1.556048

[3] Berardi U (2016) The outdoor microclimate benefits and energy saving resulting from green roofs retrofits. Energy Buildings 121:217–229. 10.1016/j.enbuild.2016.03.021

[4] Bruse M (1999) Modelling and strategies for improved urban climates. In: Proceedings international conference on urban climatology & international congress of biometeorology, pp 8–12

[5] Díaz SM, Settele J, Brondízio E, Ngo H, Guèze M, Agard J, Arneth A, Balvanera P, Brauman K, Butchart S, Chan K (2019) The global assessment report on biodiversity and ecosystem services: summary for policy makers

[6] Dudareva N, Piechersky E (2006) Floral scent metabolic pathways: their regulation and evolution. In: Dudareva N, Pichersky E (eds) Biology of floral scent. CRC Press, Boca Raton. 10.1201/9781420004007-3

[7] Free JB (1987) Pheromones of social bees. Cornell University Press, Ithaca

[8] Fuentes JD, Chamecki M, Roulston TA, Chen B, Pratt KR (2016) Air pollutants degrade floral scents and increase insect foraging times. Atmos Environ 141:361–374. 10.1016/j.atmosenv.2016.07.002

[9] Hong Kong Agriculture, Fisheries and Conservation Department (HKAFCD) (2020) https://www.afcd.gov.hk/english/conservation/hkbiodiversity/speciesgroup/speciesgroup_butterflies.html

[10] Hong Kong Development Bureau (HKDB) (2016) Parks, country and marine parks. Retrieved 2016, from https://www.greening.gov.hk/en/departments_greening_efforts/parks.html. Accessed 15 Jan 2021

[11] Hong Kong Environment Bureau (HKEB) (2016) Hong Kong biodiversity strategy action plan 2016-2021. Retrieved from: http://www.afcd.gov.hk/english/conservation/Con_hkbsap/files/HKBSAP_ENG_2.pdf. Accessed 6 Dec 2019

[12] Hong Kong Environmental Protection Department (HKEPD) (2021) Data & statistics. Retrieved from https://www.epd.gov.hk/epd/english/environmentinhk/air/data/air_data.html

[13] Kearns CA, Inouye DW (1997) Pollinators, flowering plants, and conservation biology. Bioscience 47(5):297–307. 10.2307/1313191

[14] Klein AM, Vaissiere BE, Cane JH, Steffan-Dewenter I, Cunningham SA, Kremen C, Tscharntke T (2007) Importance of pollinators in changing landscapes for world crops. Proc R Soc B Biol Sci 274(1608):303–313 10.1098/rspb.2006.3721 PMC1702377 17164193

[15] Kopec K, Burd LA (2017) Pollinators in peril: a systematic status review of north American and Hawaiian native bees. Center for Biological Diversity. https://www.biologicaldiversity.org/campaigns/native_pollinators/pdfs/Pollinators_in_Peril.pdf. Accessed 15 Jan 2021.

[16] Millennium Ecosystem Assessment (MEA) (2005) Ecosystems and human well-being: synthesis. Island Press, Washington, DC

[17] Oliver TH, Heard MS, Isaac NJ, Roy DB, Procter D, Eigenbrod F, Freckleton R, Hector A, Orme CDL, Petchey OL, Proença V (2015) Biodiversity and resilience of ecosystem functions. Trends Ecol Evol 30(11):673–684. 10.1016/j.tree.2015.08.009 26437633 10.1016/j.tree.2015.08.009

[18] O’Toole C (1993) Diversity of native bees and agroecosystems. In: Hymenoptera and biodiversity. CAB International, Wallingford, pp 169–196

[19] Potts SG, Biesmeijer JC, Kremen C, Neumann P, Schweiger O, Kunin WE (2010) Global pollinator declines: trends, impacts and drivers. Trends Ecol Evol 25(6):345–353. 10.1016/j.tree.2010.01.007 20188434 10.1016/j.tree.2010.01.007

[20] Reinhard J, Srinivasan MV (2009) The role of scents in honey bee foraging and recruitment. In: Food exploitation by social insects: ecological, behavioral, and theoretical approaches, vol 1, pp 165–182

[21] Wang Z, Wang W, Tham YJ, Li Q, Wang H, Wen L, Wang T (2017) Fast heterogeneous N2O5 uptake and ClNO2 production in power plant plumes observed in the nocturnal residual layer over the North China Plain

[22] Yan C, Tham YJ, Zha Q, Wang X, Xue L, Dai J, Wang T (2019) Fast heterogeneous loss of N2O5 leads to significant nighttime NOx removal and nitrate aerosol formation at a coastal background environment of southern China. Sci Total Environ 677:637–647. 10.1016/j.scitotenv.2019.04.389 31071666 10.1016/j.scitotenv.2019.04.389

[23] Yun H, Wang Z, Zha Q, Wang W, Xue L, Zhang L, Wang T (2017) Nitrous acid in a street canyon environment: sources and contributions to local oxidation capacity. Atmos Environ 167:223–234. 10.1016/j.atmosenv.2017.08.018

